# Bioengineered Nisin A Derivatives with Enhanced Activity against Both Gram Positive and Gram Negative Pathogens

**DOI:** 10.1371/journal.pone.0046884

**Published:** 2012-10-08

**Authors:** Des Field, Maire Begley, Paula M. O’Connor, Karen M. Daly, Floor Hugenholtz, Paul D. Cotter, Colin Hill, R. Paul Ross

**Affiliations:** 1 Department of Microbiology, University College Cork, Cork, Ireland; 2 Teagasc, Moorepark Food Research Centre, Fermoy, Co. Cork, Ireland; 3 Alimentary Pharmabiotic Centre, University College Cork, Cork, Ireland; Auburn University, United States of America

## Abstract

Nisin is a bacteriocin widely utilized in more than 50 countries as a safe and natural antibacterial food preservative. It is the most extensively studied bacteriocin, having undergone decades of bioengineering with a view to improving function and physicochemical properties. The discovery of novel nisin variants with enhanced activity against clinical and foodborne pathogens has recently been described. We screened a randomized bank of nisin A producers and identified a variant with a serine to glycine change at position 29 (S29G), with enhanced efficacy against *S. aureus* SA113. Using a site-saturation mutagenesis approach we generated three more derivatives (S29A, S29D and S29E) with enhanced activity against a range of Gram positive drug resistant clinical, veterinary and food pathogens. In addition, a number of the nisin S29 derivatives displayed superior antimicrobial activity to nisin A when assessed against a range of Gram negative food-associated pathogens, including *E. coli*, *Salmonella enterica* serovar Typhimurium and *Cronobacter sakazakii.* This is the first report of derivatives of nisin, or indeed any lantibiotic, with enhanced antimicrobial activity against both Gram positive and Gram negative bacteria.

## Introduction

**Figure 1:**
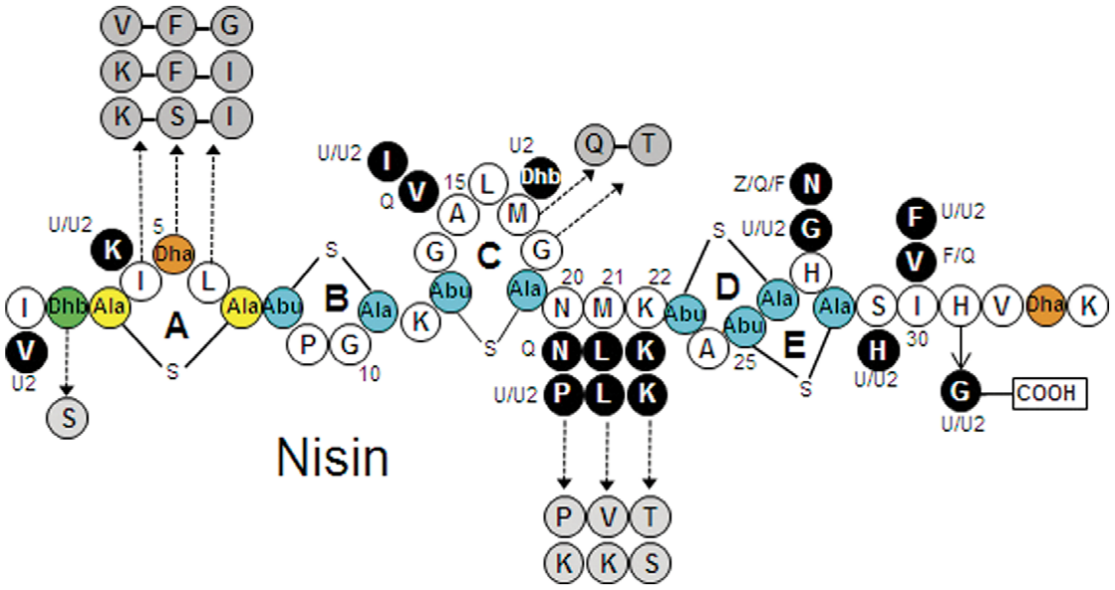
Structures of natural nisin and enhanced bioengineeredvariants. Six natural variants are known, nisin A, Z, F, Q, U and U2. Black circles indicate amino acid differences between the natural nisin variants. Broken arrows denote enhanced activity of bioengineered nisin A or Z as a result of single amino acid alterations [28], [29], [52], and/or combination of amino acid substitutions (joined circles) [22]. Residues are represented in the single letter code. Post translational modifications are indicated as follows, Dha: dehydroalanine, Dhb: dehydrobutyrine, Abu: 2-aminobutyric acid, Ala-S-Ala: lanthionine, Abu-S-Ala: 3-methyllanthionine. Adapted from [14].

Nisin is the most important commercially exploited member of the heterogeneous family of bacteriocins, antimicrobial peptides produced by bacteria that can kill or inhibit the growth of other bacteria [1]. It is the most highly characterized member of about 60 or so Class 1 bacteriocins, also termed lantibiotics. These are characterized by the presence of post-translationally modified unusual amino acids including lanthionine and/or methyllanthionine. These unusual residues are generated by a series of enzyme-mediated modifications that confer a distinct structure and stability. Many lantibiotics, including nisin, lacticin 3147 and mersacidin, are extremely potent and are active against a range of Gram positive targets including antibiotic resistant pathogens [2]–[6] as well as important food pathogen and spoilage organisms [7], [8]. Many lantibiotics are produced by lactic acid bacteria, industrially important food microorganisms that are classified as generally regarded as safe. Several have also been found to function by targeting the essential precursor of the bacterial cell wall, lipid II [9], [10], which is also a target for at least four different classes of antibiotic, including the glycopeptide vancomycin. A key advantage of lantibiotics over classical antibiotics is that they are gene-encoded and are thus much more amenable to bioengineering-based strategies with a view to further enhancing their capabilities. Indeed, bioengineering of lantibiotics has been underway for over two decades (for reviews see [11]–[14] and has provided a considerable insight into the structure and function of these peptides. It is only in recent years that researchers, armed with a greater understanding of lantibiotic biology and the application of bioengineering strategies on a larger-scale, have achieved notable successes with regard to enhancing the antimicrobial activity of lantibiotics against pathogenic bacteria. Both mersacidin and nukacin have been the subject of comprehensive site-saturation mutagenesis approaches which have resulted in the generation of several novel derivatives with enhanced activity compared to the parent peptide [15], [16]. In the case of mersacidin, this included variants with enhanced activity against methicillin resistant *Staphylococcus aureus* (MRSA), vancomycin resistant enterococci (VRE) and *S. pneumonia*. Nisin itself has been subjected to bioengineering for almost twenty years [17]–[26]. For a comprehensive overview of the data generated by these studies the reader is directed to a number of reviews on the topic ([12]–[14], [27]. Despite the large collection of derivatives which have been generated, relatively few have been found to exhibit enhanced activity against pathogenic microorganisms. An obvious exception relates to derivatives generated by targeting a short 3 amino acid stretch (Asn20-Met21-Lys22) in the centre of the peptide, known as the ‘hinge-region’. Initial success was achieved through the generation of two mutants, N20K and M21K (Fig. 1), which displayed enhanced activity against Gram negative bacteria including *Shigella*, *Pseudomonas* and *Salmonella* spp. [28]. The generation of nisin derivatives with enhanced activity against Gram positive pathogens was achieved 4 years later using a non-targeted approach [29]. In this instance, the use of a random mutagenesis-based approach to create approximately 8000 nisin derivatives led to the identification of one variant, K22T (Fig. 1), that displayed enhanced activity against *Streptococcus agalactiae*, a human and bovine pathogen. Prompted by the identification of yet another ‘hinge’ derivative with enhanced activity, a more extensive ‘hinge-specific’ mutagenesis strategy was undertaken. This led to the identification of further derivatives of note. Four of these were selected for closer inspection [29], with M21V (Fig. 1) being particularly notable by virtue of its enhanced antimicrobial activity against a wide range of targets, including medically significant pathogens such as heterogenous Vancomycin intermediate *Staphylococcus aureus* (hVISA), VRE, MRSA, *Clostridium difficile*, *S. agalactiae* and *Listeria monocytogenes* [29], [30]. This enhanced activity was also apparent in a food setting [30]. Studies with nisin K22T (nisin T) revealed it to be more potent than nisin A against veterinary isolates of *S. aureus, S. agalactiae* [30] and *M. tuberculosis* [31] while N20P (nisin P) (Fig. 1) is noteworthy by virtue of the target specific nature of its enhanced activity [29]. More recently, a number of additional nisin ‘hinge’ derivatives have also been identified which exhibit enhanced activity relative to nisin A in complex matrices [32].

Prompted by the success of the aforementioned random mutagenesis approach, we further screened the bank of randomly generated nisin derivatives using additional target species not included in previous studies (*S. aureus* SA113 and *L. monocytogenes* LO28). One derivative (S29G) displayed enhanced activity against *S. aureus* SA113. S29G was subjected to complete saturation mutagenesis to investigate the impact of replacing serine with all 19 other standard amino acids on the bioactivity of nisin. The results reveal the importance of position 29 with respect to the activity of nisin and have for the first time led to the identification of derivatives with enhanced activity against both Gram positive and Gram negative pathogens.

## Materials and Methods

### Bacterial Strains and Growth Conditions

The bacterial strains used in this study are listed in Table 1. *L. lactis* strains were grown in M17 broth supplemented with 0.5% glucose (GM17) or GM17 agar at 30°C. *S. aureus* strains were grown in Mueller-Hinton (MH) broth (Oxoid) or MH agar at 37°C, streptococci and *Bacillus* strains were grown in Tryptic soy broth (TSB) or TSB agar at 37°C, *Listeria* strains were grown in Brain Heart Infusion (BHI) or BHI agar at 37°C. *C. sakazakii, E. coli* and *Salmonella* strains were grown in Luria-Bertani broth with vigorous shaking or agar at 37°C unless otherwise stated. Antibiotics were used where indicated at the following concentrations: Chloramphenicol at 10 and 20 µg ml^−1^, respectively for *L. lactis* and *E. coli.* Tetracycline was used at 10 µg ml^−1^for *L. lactis* and *E. coli.*

**Table 1:** Strains used in this study.

Strains	Relevant characteristics	Reference
*L. lactis* NZ9700	Wild type Nisin producer	[53], [54]
*L. lactis* NZ9800	*L. lactis* NZ9700Δ*nis*A	[53], [54]
*L. lactis* NZ9800pDF05	*L. lactis* NZ9800 harboring pCI372 with nisA under its own promoter	[29]
*L. lactis* NZ9800pDF03	*L. lactis* NZ9800 harboring pPTPL with nisA under its own promoter	[29]
*E. coli* Top10	Intermediate cloning host	Invitrogen
*E. coli* MC1000	*E. coli* host for pPTPL	[55]
**Indicator organisms**		
*Strep. agalactiae* ATCC13813	Nisin sensitive indicator	ATCC
*Strep. mitis* UCC5001	Nisin sensitive indicator	UCC Culture Collection
*S. aureus* RF122	Nisin sensitive indicator	DPC Collection
*S. aureus* Sa113	Nisin sensitive indicator	UCC Culture Collection
ST 528^a^	Nisin sensitive indicator	BSAC
ST 530^a^	Nisin sensitive indicator	BSAC
hVISA 32679^b^	Nisin sensitive indicator	BSAC
*L. monocytogenes* 10403S	Nisin sensitive indicator	UCC Culture Collection
*L. monocytogenes* LO28	Nisin sensitive indicator	UCC Culture Collection
*B. cereus* DPC 6088	Nisin sensitive indicator	DPC Collection
*B. cereus* DPC 6089	Nisin sensitive indicator	DPC Collection
*L. lactis* spp cremoris HP	Nisin sensitive indicator	UCC Culture Collection
*L. lactis* MG1363	Nisin sensitive indicator	UCC Culture Collection
*E. durans* 5133	Nisin sensitive indicator	DPC Collection
*E. coli* 0157-H7	Nisin sensitive indicator	UCC Culture Collection
*C. sakazakii* DPC 6440	Nisin sensitive indicator	DPC Collection
*Salmonella enterica* serovar Typhimurium UK1	Nisin sensitive indicator	UCC Culture Collection

DPC - Dairy Products Research Centre, BSAC – British Society for Antimicrobial Chemotherapy, ATCC – American Type Culture Collection,

a Methicillin resistant *S. aureus*.

b heterogenous Vancomycin-intermediate *S. aureus*.

### Random Mutagenesis

DNA obtained from *L. lactis* NZ9700 [33] was used as template for the amplification of a 372 bp fragment encompassing the *nis*A gene with KOD polymerase (Novagen) using the primers oDF101 and oDF102 (Table 2). PCR amplicons were purified using the QIAquick PCR purification kit (QIAGEN Inc.), digested with *Bgl*II and *Xba*I (Roche) and cloned into similarly digested and Shrimp Alkaline Phosphatase (SAP; Roche) treated pPTPL. Following introduction into *E. coli* MC1000, plasmid was isolated from one clone and was sequenced (MWG Biotech, Germany) using the primer TETK P1 to ensure its integrity. The introduction of this plasmid, pDF03, into competent *L. lactis* NZ9800 successfully reinstated nisin activity. To provide sufficient quantities of template DNA for error-prone PCR (ep-PCR), *nis*A was reamplified using pDF03 as template with KOD polymerase using the primers oDF101 and oDF103, digested with *Xba*1 and *Eco*R1 and cloned into similarly digested pUC19. Following introduction into *E. coli* Top 10 (Invitrogen), plasmid was isolated from one clone and was sequenced (MWG Biotech, Germany) using the primers M13FOR and M13REV to ensure its integrity. This plasmid, pDF04 was isolated from 100 ml overnight culture using the Maxi-prep plasmid kit (QIAGEN Inc.) to a concentration of approx 1,100 ng/µl. pDF04 was used as template for the Genemorph II random mutagenesis kit (Stratagene) according to manufacturer’s guidelines. To introduce an average of one base change in the 372 bp cloned fragment, amplification was performed in a 50 µl reaction containing approximately 500 ng of target DNA (pDF04), 2.5 units Mutazyme DNA polymerase, 1 mM dNTPs and 200 ng each of primers oDF101 and oDF102. The reaction was preheated at 96°C for 1 min, and then incubated for 22 cycles at 96°C for 1 min, 52°C for 1 min and 72°C for 1 min, and then finished by incubating at 72°C for 10 min. Amplified products were purified by gel extraction using the Qiaquick gel extraction kit (QIAGEN Inc), and reamplified with KOD polymerase before being digested with *Bgl*II and *Xba*I (Roche), ligated with similarly digested and SAP treated pPTPL and introduced into *E. coli* MC1000. To determine if the correct rate of mutation had been achieved recombinant plasmid DNA was isolated from selected clones using the QIAprep Spin miniprep kit (QIAGEN Inc) and sequenced (MWG Biotech). Transformants were pooled and stored in 80% glycerol at −20°C. Plasmid DNA isolated from the mutant bank was used to transform *L. lactis* NZ9800. Transformants (approx. 8000) were isolated from Q trays using the Genetix QPIX II-XT colony-picking robot and inoculated into 96 well plates containing GM17 freezing buffer, incubated overnight and subsequently stored at −20°C.

**Table 2:** Oligonucleotides used in this study.

Primer name	Sequence
oDF101	5′TCAGATCTTAGTCTTATAACTATACTG 3′
oDF102	5′ TGTCTAGATTATTTGCTTACGTGAATA 3′
oDF103	5′ CGGAATTCTAGTCTTATAACTATAGTGA 3′
oDF105	5′ AACTGCAGTATAGTTGACGAATA 3′
oDF106	5′ TAGAATTCAACAGACCAGCATTA 3′
M13FOR	5′ GTAAAACGACGGCCAGTG 3′
M13REV	5′ GGAAACAGCTATGACCATG 3′
NisS29degFOR	5′ Pho-TGTCATTGT**NNK**ATTCACGTAAGCAAATAA 3′
NisS29degREV	5′ TACGTGAAT**MNN**ACAATGACAAGTTGCTGTTTTCATGTT 3′
NisS29QFOR	5′ Pho- TGT CAT TGT *CAG* ATT CAC GTA AGC AAA TAA
NisS29QREV	5′ TAC GTG AAT *CTG* ACA ATG ACA AGT TGC TGT TTT CAT GTT
pCI372FOR	5′- CGGGAAGCTAGAGTAAGTAG -3′
pCI372REV	5′- ACCTCTCGGTTATGAGTTAG -3′

Underlined sequences represent restriction sites. Boldface represents randomised nucleotides (N = A+C+G+T, K = G+T, M = A+C). Italics represent altered codons.

### Saturation Mutagenesis

To generate a template for mutagenesis, the 372 base pair fragment encompassing the nisA gene was amplified with KOD polymerase using the primers oDF102 and oDF103, was digested and subsequently cloned into pCI372. Following introduction into E. coli Top 10 cells, plasmid was isolated from one clone and was sequenced (MWG Biotech, Germany) using the primer pCI372REV to ensure its integrity. Saturation mutagenesis of the serine codon at position 29 of *nis*A was carried out with pDF05 (pCI372-*nis*A) as template and using oligonucleotides NisS29degFOR and NisS29degREV (Table 2) containing an NNK codon in place of each native codon. PCR amplification was performed in a 50 µl reaction containing approximately 0.5 ng of target DNA (pDF05), 1 unit Phusion High-Fidelity DNA polymerase (Finnzymes, Finland), 1 mM dNTPs and 500 ng each of the appropriate forward and reverse oligonucleotide. The reaction was preheated at 98°C for 2 mins, and then incubated for 29 cycles at 98°C for 30 secs, 55°C for 15 secs and 72°C for 3 mins 30 secs, and then finished by incubating at 72°C for 3 mins 30 secs. Amplified products were treated with Dpn1 (Stratagene) for 60 mins at 37°C to digest template DNA and purified using the QIAquick PCR purification kit. Following transformation of *E. coli* Top 10 cells plasmid DNA was isolated and sequenced using the primers pCI372FOR and pCI372REV (Table 2) to verify that mutagenesis had taken place. The purified products were subsequently introduced by electroporation into the strain NZ9800 which has all the genes necessary for nisin production. Approximately 180 transformants were chosen at random and inoculated into 96 well plates containing GM17 chloramphenicol, incubated overnight and stored at −20°C after addition of 80% glycerol.

### Site-directed Mutagenesis to Obtain S29Q

To obtain the last remaining unidentified variant S29Q, site-directed mutagenesis was undertaken using the oligonucleotides nisS29QFor and nisS29QRev (Table 2). Approximately 0.5 ng of the plasmid pDF05 (pCI372-nisA) was used as template for the PCR reaction which was performed in a 50 µl reaction containing 1 unit Phusion High-Fidelity DNA polymerase (Finnzymes, Finland), 1 mM dNTPs and 500 ng each of the appropriate forward and reverse oligonucleotide. The reaction was preheated at 98°C for 2 mins, and then incubated for 29 cycles at 98°C for 30 secs, 55°C for 15 secs and 72°C for 3 mins 30 secs, and then concluded by incubating at 72°C for 3 mins 30 secs. Amplified products were treated with Dpn1 (Stratagene) for 60 mins at 37°C to digest template DNA and purified using the QIAquick PCR purification kit. The purified products were subsequently introduced by electroporation into the strain NZ9800. Transformants were subjected to mass spectrometry to identify peptides with a mass corresponding to 3394 amu indicative of the desired S29Q mutation.

### Nisin Purification

*L. lactis* NZ9700 (nisin A producer) or the mutant nisin strain of interest was subcultured twice in GM17 broth at 1% at 30°C before use. Two litres of modified TY broth were inoculated with the culture at 0.5% and incubated at 30°C overnight. The culture was centrifuged at 7,000 rpm for 15 minutes. The cell pellet was resuspended in 300 mls of 70% 2-propanol 0.1% trifluoroacetic acid (TFA) and stirred at room temperature for approximately 3h. The cell debris was removed by centrifugation at 7,000 rpm for 15 minutes and the supernatant retained. The 2-propanol was evaporated using a rotary evaporator (Buchi) and the sample pH adjusted to 4 before applying to a 10g (60 ml) Varian C-18 Bond Elut Column (Varian, Harbor City, CA) pre-equilibrated with methanol and water. The columns were washed with 100 mls of 20% ethanol and the inhibitory activity was eluted in 100 mls of 70% 2-propanol 0.1% TFA. 15 ml aliquots were concentrated to 2 ml through the removal of 2-propanol by rotary evaporation. 1.5 ml aliquots were applied to a Phenomenex (Phenomenex, Cheshire, UK) C12 reverse phase (RP)-HPLC column (Jupiter 4u proteo 90 Å, 250×10.0 mm, 4 µm) previously equilibrated with 25% 2-propanol, 0.1% TFA. The column was subsequently developed in a gradient of 30% 2-propanol containing 0.1% TFA to 60% 2-propanol containing 0.1% TFA from 10 to 45 minutes at a flow rate of 1.2 ml min^−1^.

### Mass Spectrometry

For Colony Mass Spectrometry (CMS) bacterial colonies were collected with sterile plastic loops and mixed with 50 µl of 70% isopropanol adjusted to pH 2 with HCl. The suspension was vortexed, the cells centrifuged in a benchtop centrifuge at 14,000 r.p.m. for 2 mins, and the supernatant was removed for analysis. Mass Spectrometry in all cases was performed with an Axima CFR plus MALDI TOF mass spectrometer (Shimadzu Biotech, Manchester, UK). A 0.5 µl aliquot of matrix solution (alpha-cyano-4-hydroxy cinnamic acid (CHCA), 10 mg ml^−1^ in 50% acetonitrile-0.1% (v/v) trifluoroacetic acid) was placed onto the target and left for 1–2 mins before being removed. The residual solution was then air-dried and the sample solution (resuspended lyophilised powder or CMS supernatant) was positioned onto the precoated sample spot. Matrix solution (0.5 µl) was added to the sample and allowed to air-dry. The sample was subsequently analysed in positive-ion reflectron mode.

### Bioassays for Antimicrobial Activity

Deferred antagonism assays were performed by replicating strains on GM17 agar plates and allowing them to grow overnight before overlaying with either GM17/BHI/TSB-YE/MH agar (0.75% w/v agar) seeded with the appropriate indicator strain. For higher throughput screening of the S29X bank, deferred antagonism assays were performed by replicating strains using a 96 pin replicator (Boekel) or spotting 5 µl of a fresh overnight culture on GM17 agar plates and allowing them to grow overnight. Following overnight growth the strains were subjected to UV radiation for 30 minutes prior to overlaying with either GM17/BHI/TS/MH agar (0.75% w/v agar) seeded with the appropriate indicator.

Agarose-based deferred antagonism assays were carried out as follows: GM17/LB (0.03%) underlay was prepared with 10 mM sodium phosphate buffer (SPB) at pH 7.4 to which was added agarose (1% w/v agarose), autoclaved and cooled to 50°C. Bacteria grown to mid-logarithmic phase were harvested by centrifugation and washed with 10 mM SPB at pH 7.4. Bacteria were then added to 15 mls cooled underlay medium to reach a concentration of 2×10^7^ colony-forming units (CFU)/mL. The inoculated medium was rapidly transferred into sterile Petri plates, allowed to solidify and dried. Wells (4.6 mm in diameter) were then made in the seeded plates. 10 µl volumes of cell-free supernatant from overnight nisin derivative producing cultures or resuspended purified peptides were then added to the wells and the plates incubated at 30°C (*L. lactis*) or 37°C (Gram positive strains) or RT (Gram negative strains) for 3 hours. Polymyxin B (Sigma Aldrich) was used at a concentration of 20 µg/ml. The plates were then overlaid with 15 mls of autoclaved double strength GM17 (*L. lactis*) or LB (Gram negatives) agarose (1% w/v agarose) overlay medium precooled to 50°C. The plates were then incubated overnight at the relevant temperature.

### Minimum Inhibitory Concentration Assays

Minimum inhibitory concentration determinations for Gram positive organisms were carried out in triplicate in microtitre plates (Sarstedt). 96 well microtitre plates were pre-treated with bovine serum albumin (BSA) prior to addition of the peptides. Briefly, to each well of the microtitre plate 200 µL of phosphate buffered saline (PBS) containing 1% (w/v) bovine serum albumin (PBS/BSA) was added and incubated at 37°C for 30 min. The wells were washed with 200 µL PBS and allowed to dry. Target strains were grown overnight in the appropriate conditions and medium, subcultured into fresh broth and allowed to grow to an OD_600_ of ∼0.5, diluted to a final concentration of 10^5^ cfu ml^−1^ in a volume of 0.2 ml. Wild type nisin and nisin mutant peptides were adjusted to a 10 µM (*S. mitis, B. cereus*), 7.5 µM (*L. monocytogenes*), 5 µM (hVISA) 2.5 µM (MRSA, *E. durans*), or 500 nM (*L. lactis* strains) starting concentration and 2-fold serial dilutions of each peptide were added to the target strain. After incubation for 16 h at 37°C the MIC was read as the lowest peptide concentration causing inhibition of visible growth.

Minimum inhibitory concentration determinations for Gram negative strains were carried out in triplicate in 96 well microtitre plates. Briefly, bacteria (*E. coli*, *C. sakazakii* and *Salmonella*) grown to mid-logarithmic phase were harvested by centrifugation, washed with 10 mM SPB at pH 7.4, and diluted to 2×10^5^ colony-forming units (CFU)/mL in SPB. Nisin and nisin derivative peptides were resuspended in sterile HPLC water and 50 µL aliquots were added to wells containing 50 µL of 2×10^5^ CFU of bacteria. Plates were incubated at RT for two hours with agitation. Double strength Luria–Bertani (LB) broth (100 µL) was added and the plates were incubated at RT overnight, the MIC was taken as the lowest concentration at which growth was inhibited.

## Results

### Screening a Bank of Random Nisin Peptides

**Figure 2:**
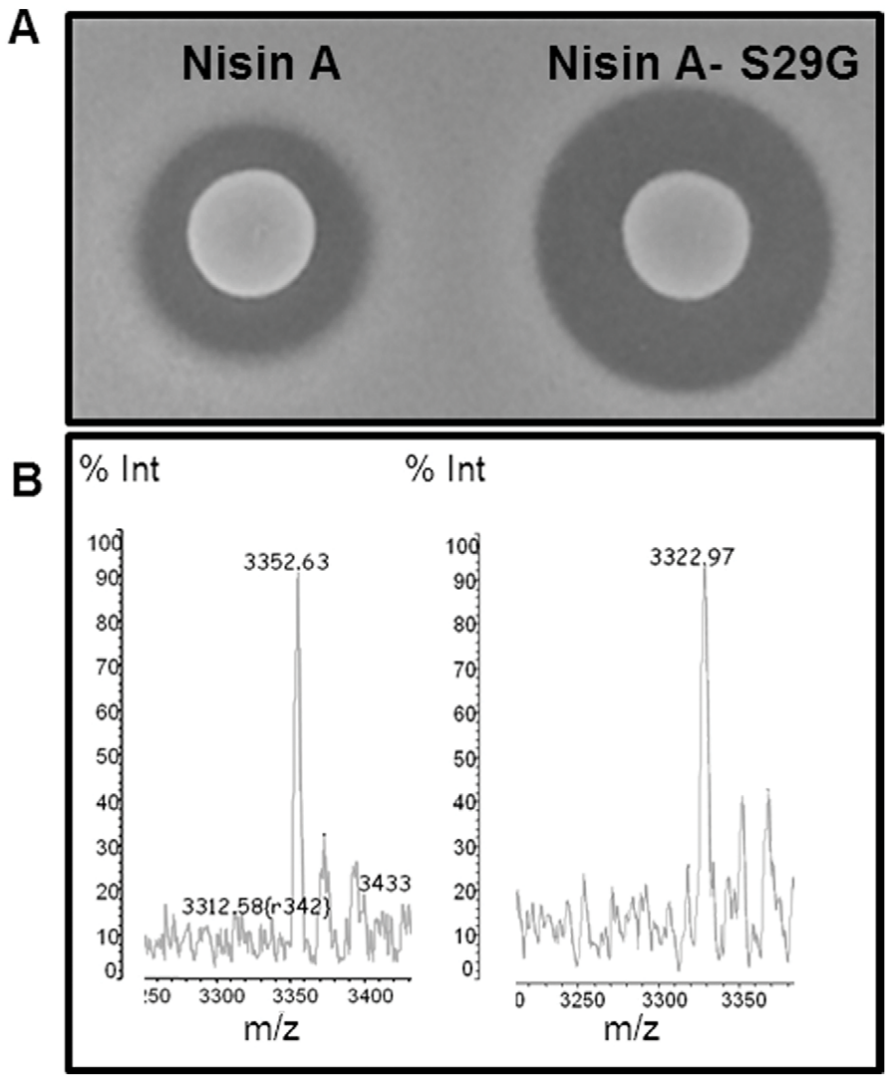
Bioactivity and mass spectrometry analysis of nisin A and nisin A S29G. Growth inhibition of (A) *S. aureus* SA113 by the nisin A producing strains NZ9800pPTPL-*nisA* and NZ9800pPTPL-*nisA* S29G (B) Colony Mass Spectrometry analysis of nisin A (3352.63 amu) and the nisin A S29G (3322.97 amu) derivative.

Although random mutagenesis approaches have rarely been applied to lantibiotics, the recent randomization of the nisin A structural gene led to the identification of a beneficial change with respect to anti-Gram positive activity [29]. Follow-up site-directed and site-saturation mutagenesis within the same region, corresponding to the ‘hinge’ of the peptide, identified three further beneficial mutations with respect to antimicrobial activity [29]. Given that only two indicators (*L. lactis* HP and *S. agalactiae* ATCC13813) were employed during the original screen of the random bank of >8,000 nisin-producing derivatives, a decision was made to re-screen this bank using *S. aureus* SA113 and *L. monocytogenes* LO28 as targets. The strategy proved successful in that it revealed one isolate that displayed superior bioactivity against *S. aureus* SA113 (Fig. 2), but which exhibited an activity comparable to that of nisin A against *L. monocytogenes* LO28 (data not shown). Bioactivity refers to the zone of inhibition surrounding a producer colony, or the zone created in a deferred antagonism assay. This could be a result of increased specific activity, enhanced solubility or even enhanced production, but serves as a useful first step in identifying beneficial changes. The enhanced isolate was selected for closer inspection. A peptide mass of 3322.97 Da (Fig. 2) determined by colony mass spectrometry (CMS) suggested a S29G alteration had occurred in the mature peptide. This was in agreement with DNA sequence analysis which confirmed that the serine at position 29 was altered. This is an unusual residue in that it is the only potentially modifiable residue (serine, threonine or cysteine) in nisin A that remains unmodified. It is also the first residue of a 6 amino acid stretch directly following rings D and E located in the C-terminus of nisin (Fig. 1), which is thought to insert into target membranes to form pores [34].

### Creation and Analysis of a Bank of Nisin A Serine29 Derivatives

**Figure 3:**
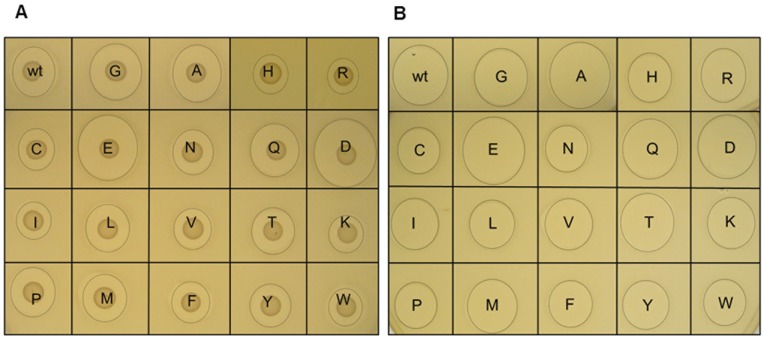
Deferred antagonism assays of nisin A S29 derivatives against the nisin-sensitive indicator *Lactococcus lactis* HP. (A) spot on lawn of producing strains on GM17 agar and overlaid with GM17 agar (0.75%) seeded with HP and (B) supernatants of producing strains in agarose-based (1%) GM17. Single letters correspond to IUPAC abbreviation code, wt = Serine.

Serine 29 has previously been shown to be an important residue with respect to activity (truncated versions of nisin, nisin 1–29 and nisin 1–28, display 16 and 100 fold reduced activity, respectively [35], [36]. We decided that further targeting of this position was warranted. A saturation mutagenesis approach was undertaken, similar to the strategy carried out previously [29]. This involves using oligonucleotides to replace the specific codon (in this case the AGT coding for serine 29) with an NNK triplet, potentially encoding all 20 standard amino acids. Complete plasmid amplification of pDF05 (*nisA* cloned into a shuttle vector, pCI372) was carried out and the products were transformed into an *E. coli* Top10 host. Following plasmid extraction, the pooled bank of pDF05 derivatives was introduced into *L. lactis* NZ9800 to allow expression of the mutant nisin A peptides for further analysis. The bioactivity of approximately 200 *L. lactis* NZ9800 pDF05 derivatives was assessed using deferred antagonism assays against a range of target indicator organisms including *S. aureus* RF122, *L. monocytogenes* LO28, *S. agalactiae* ATCC13813 and *L. lactis* ssp *cremoris* HP. In addition, the same derivatives were analyzed by Mass Spectrometry to identify the extent and nature of the amino acid substitutions which had occurred. The strategy proved highly successful in that 19 of the potential 20 alterations were detected (Table 3). To complete the collection, site-directed mutagenesis was employed to create the final derivative, S29Q. Screening of this collection of Ser29 derivatives revealed that a number of derivatives, or the supernatants they produced, exhibited superior activity to nisin A producers in agar-based deferred antagonism assays and agarose-based antimicrobial assays against *L. lactis* HP (Fig. 3), *S. aureus* RF122 and *S. agalactiae* ATCC13813 (data not shown). The bioactivity of the S29G variant generated was enhanced as expected, while three additional variants also exhibited enhanced activity against at least one target. These derivatives produced peptides with S29A, S29D and S29E changes (Fig. 3A), as confirmed by mass spectrometry and DNA sequencing.

**Table 3:** MALDI TOF mass spectrometry analysis of nisin S29 saturation derivatives corresponding to all 20 standard amino acids.

Amino acid		Molecular Mass	
S29X		Predicted	Actual
Asparagine	N	3379.66	3379.05
Glutamine	Q	3393.69	3393.23
Cysteine	C	3368.70	3368.73
Glycine	G	3322.60	3322.97
Alanine	A	3336.64	3336.67
Serine	S	3352.06	3352.63
Threonine	T	3366.66	3366.75
Valine	V	3364.69	3364.81
Leucine	L	3378.71	3379.33
Isoleucine	I	3378.71	3378.60
Proline	P	3362.67	3362.73
Methionine	M	3396.74	3396.15
Phenylalanine	F	3412.73	3412.94
Tyrosine	Y	3428.73	3429.17
Tryptophan	W	3451.76	3451.61
Aspartic acid	D	3380.64	3381.32
Glutamic acid	E	3394.67	3395.56
Arginine	R	3421.74	3421.93
Histidine	H	3402.69	3404.15
Lysine	K	3393.72	3394.73

These assays also provided valuable information regarding the consequences of incorporating other residues at this location. These consequences can be grouped according to the nature of the newly incorporated residue and, in this instance, were compared in terms of their relative impact on bioactivity against *L. lactis* HP (Table 4). Nisin A is naturally devoid of aromatic residues, and all bioengineered derivatives in which aromatic residues have been incorporated have displayed reduced antimicrobial activity (I1W, M17W, N20F, N20Y, N20W, M21F, M21Y, M21W, K22F, K22W, V32W, I30W, and N20F/M21L/K22Q [28], [37], [38]. This pattern is again apparent from our studies in that S29F, S29Y and S29W changes were all found to impact negatively on bioactivity (Table 4).

**Table 4:** Agarose assay results of S29 derivatives against *Lactococcus lactis* HP.

	Nisin derivative (S29X)	Zone Diameter (mm)	% difference (P value)
**Hydrophillic : Neutral**	Serine (wt)	15.2±0.2	100
	Threonine (T)	12.1±0.9	79
	Glutamine (Q)	15.3**±**0.1	100
	Asparagine (N)	12.6**±**1.0	83
	Tyrosine (Y)	10.6±0.6	70
**Hydrophillic : Charged**	Aspartate (D) −ve	**20.3±0.8**	**133 (0.006)**
	Glutamate (E) −ve	**18.6±0.3**	**122 (0.0004)**
	Arginine (R) +ve	11.0**±**0.6	72
	Histidine (H) +ve	10.9**±**0.3	72
	Lysine (K) +ve	9.9**±**1.0	64
**Hydrophobic :**	Alanine (A)	**16.6±0.3**	**109 (0.004)**
	Valine (V)	11.6±0.4	76
	Glycine (G)	15.4±0.5	101
	Cysteine	10.5**±**1.0	69
	Leucine (L)	12.7±0.7	84
	Isoleucine (I)	9.9±1.0	65
	Tryptophan (W)	12.1±1.2	79
	Phenylalanine (F)	11.0±1.0	72
	Methionine (M)	13.6**±**0.8	89
	Proline (P)	13.7**±**1.0	89

Results are expressed as zone diameter in mm. Bold font denotes activity greater than wild type nisin A. Values in bold reached statistical significance compared to a nisin control (Student’s t-test: P<0.05).

Nisin A is cationic due to the presence of 5 positively charged residues (Lys12, Lys22, Lys34, His27, His31) and the absence of negatively charged equivalents. To date, the effect of manipulating the charge of nisin has had variable outcomes. This may be a consequence of the location at which the charge residues are incorporated. For instance, the introduction of negatively charged residues into the hinge region has had a detrimental impact (N20D, N20E, M21E, K22D and K22E; [28], [29] whereas the introduction of positively charged residues has had a more beneficial impact on anti-Gram-negative activity (N20K and M21K; [28]. Given the importance of positive charge for the initial attraction of many cationic peptides to the cell envelope, it was surprising to find that the introduction of negatively charged residues (S29D, S29E) resulted in the corresponding peptides exhibiting superior bioactivity. S29D and S29E derivatives also displayed higher bioactivity than that of wild type nisin A producer against *L. lactis* HP (Table 4). In contrast, the replacement of serine 29 with positively charged residues had a negative impact in that the variants with S29R, S29H and S29K changes demonstrated reduced bioactivity.

Replacement of serine with threonine also produced a peptide with a moderate reduction in activity. The mass of this peptide was consistent with the presence of threonine in its unmodified form (3366.75 Da).

In the past, the introduction of hydrophobic residues have had varied impacts on the bioactivity of nisin. In the case of the hinge-region, the introduction of isoleucine, leucine, and methionine (in the latter case at positions 20 or 22) resulted in decreased bioactivity in the majority of cases. In contrast, the introduction of a proline at Asn20 (N20P) or a valine at Met21 (M21V) resulted in peptides with enhanced activity [29], [30]. In the present study, the incorporation of isoleucine, leucine, proline and methionine at position 29 resulted in modest reductions in bioactivity against *L. lactis* HP (Table 4).

The impact on bioactivity as a result of the incorporation of small and nucleophilic residues has generally been favorable, particularly with respect to the hinge region [29]. As noted above, the introduction of a glycine at position 29 (S29G) increases the bioactivity of the corresponding strain against *S. aureus* RF122. However, the bioactivity of this strain against *L. lactis* HP is comparable to that of nisin A (Table 4) while the incorporation of alanine (S29A) has a more beneficial impact (Table 4). Finally, the derivatives S29N and S29Q exhibited 83% and 100% of wild type activity, respectively.

As a consequence of the apparent improved bioactivity of the producers of the nisin A S29G, S29A, S29D and S29E derivatives against at least one target, these four derivatives were purified in order to determine if enhanced bioactivity was attributable to enhanced specific activity.

### Specific Activities of Nisin A S29G, S29A S29D and S29E against Gram Positive Microorganisms

Although agar-based assays are commonly used for antibiotic MIC determinations in solid media, a number of drawbacks have been identified that relate to altered diffusion rates. This is especially true for molecules of a more hydrophobic or amphiphillic nature, or ones which interact with the diffusion medium, or suffer degradation or loss of substrate during diffusion [39]. To ensure that the enhanced activity of the selected S29 derivatives was not as a consequence of altered diffusion rates, the specific activity of the peptides was assessed against a wide range of organisms using classical broth-based minimum inhibitory concentration (MIC) determination assays. Targets included the antibiotic resistant *S. aureus* strains ST 528 (MRSA), ST 530 (MRSA), hVISA 32679, as well as *S. aureus* RF122, *Streptococcus mitis*, *L. lactis* HP and MG1363, *Bacillus cereus* DPC 6088/6089, *Enterococcus durans* and *L. monocytogenes* strains 10403S and LO28.

Using equimolar concentrations of purified peptides, the specific activities of S29G, S29A, S29D and S29E were determined (Table 5). For the purpose of comparison, a M21K derivative of nisin A was also generated and purified. A M21K derivative of nisin Z was previously found to possess enhanced activity against some Gram negative targets. These investigations established that the MIC of nisin A against MRSA strains ST 528 and ST 530 was 0.5 and 0.5 mg/L, respectively (Table 5), which was in close agreement with previous results [3]. Both nisin S29G and S29A were two-fold more active than nisin A against both of these targets (0.26 mg/L in each case). One of the other serine 29 derivatives, S29D, displayed a similarly enhanced specific activity against one of these strains, MRSA ST 528. Furthermore, the S29G and S29A derivatives also exhibited improved specific activity against hVISA 32679, with MIC values of 2 mg/L and 2 mg/L, respectively, compared to an MIC of 4 mg/L for nisin A (Table 5). Against the MSSA (methicillin sensitive *S. aureus*) strain, *S. aureus* RF122, both S29G and S29A also outperformed nisin A (MICs of 0.52, 0.52 and 1.04 mg/L, respectively). However, the ‘charge’ mutants S29D and S29E were less active than wild type against this target (4.19, 2.0 and 1.04 mg/L, respectively). In contrast, S29D and S29E were found to be particularly active when *L. lactis* HP was used as the indicator organism. While the MIC for nisin A was 0.2 mg/L and that of both S29G and S29A was 0.1 mg/L, the MICs of S29D and S29E were a mere 0.05 mg/L. This pattern appeared to be strain variable in that when another *L. lactis* MG1363 strain was employed both S29G and S29D were twice as active (0.4 mg/) as nisin A whereas in this instance nisin S29A was most potent (four fold more active than nisin A (0.2 and 0.8 mg/L, respectively). In contrast, the specific activity of nisin S29E was equal to that of nisin A against *L. lactis* MG1363.

**Table 5:** Minimum inhibitory concentration results of nisin derivatives against representative Gram positive strains.

STRAIN	NisinA mg/L (µM)	S29G mg/L (µM)	S29A mg/L (µM)	S29D mg/L (µM)	S29E mg/L (µM)	M21K mg/L (µM)
ST 528^a^	0.52 (0.156)	**0.26 (0.078)**	**0.26 (0.078)**	**0.26 (0.078)**	0.52 (0.15)	nd
ST 530 a	0.52 (0.156)	**0.26 (0.078)**	**0.26 (0.078)**	0.52 (0.156)	0.52(0.15)	nd
hVISA 32679 b	4.19(1.25)	**2 (0.625)**	**2 (0.625)**	4.19(1.25)	4.19(1.25)	nd
*L.mono*10403S	12.57 (3.75)	**6.28 (1.875)**	**6.28 (1.875)**	12.57 (3.75)	12.57 (3.75)	nd
*L.mono* LO28	6.28 (1.875)	6.28 (1.875)	**3.14 (0.937)**	12.57 (3.75)	6.28 (1.875)	nd
*L. lactis* MG1363	0.8 (0.250)	**0.4 (0.125)**	**0.2 (0.062)**	**0.4 (0.125)**	0.8 (0.250)	nd
*L. lactis* HP	0.2 (0.062)	**0.1 (0.031)**	**0.1 (0.031)**	**0.05 (0.015)**	**0.05(0.015)**	0.2 (0.062)
*S. aureus* RF122	1.04 (0.312)	**0.52 (0.156)**	**0.52 (0.156)**	4.19(1.25)	2 (0.625)	1.04 (0.312)
*S. mitis* UCC5000	8.38 (2.5)	8.38 (2.5)	**4.19 (0.125)**	16.76 (5.0)	**4.19 (0.125)**	8.38 (2.5)
*B.cereus* DPC 6088	8.38 (2.5)	**4.19(1.25)**	**2 (0.612)**	16.76 (5.0)	**4.19(1.25)**	16.76 (5.0)
*B. cereus* DPC 6089	8.38 (2.5)	**4.19(1.25)**	**4.19(1.25)**	8.38 (2.5)	**4.19(1.25)**	16.76 (5.0)
*E. durans* 5133	0.52 (0.156)	**0.26 (0.078)**	**0.13 (0.039)**	0.52 (0.156)	0.52 (0.156)	0.52 (0.156)

a Methicillin resistant *S. aureus*.

b heterogenous Vancomycin-intermediate *S. aureus*. nd-not determined.

Minimum inhibitory concentration results of purified nisin wild type peptide and the S29 derivatives S29G, S29A, S29D, S29E and the hinge derivative M21K against a range of Gram positive indicators. Results are expressed as the mean of triplicate assays.

Two other foodborne associated strains were selected for MIC analysis, including isolates of *Bacillus cereus* and *Enterococcus durans. B. cereus* is ubiquitous in the environment but is often found in food production locations due to its ability to form biofilms and highly adhesive endospores, enabling it to survive food processing treatments [40]. Enterococci are present in high numbers in food of animal origin [41] and vegetables [42] and are recognized as a frequent cause of nosocomial infections [43]. The efficacies of S29G and S29E were two-fold better against *B. cereus* DPC 6088 and DPC 6089 (Table 5), while S29A was also twice as potent as nisin A against *B. cereus* DPC 6089 but displayed four-fold improvement against *B. cereus* DPC 6088. In contrast, S29G was the only derivative to display two-fold improvement against *E. durans* 5133, while S29A displayed a four-fold increase in potency to nisin A (0.13 mg/L and 0.52 mg/L, respectively (Table 5).

Interestingly, although the specific activity of S29G was enhanced against almost all Gram positive targets tested, in two instances its activity equaled nisin A (Table 5). More specifically, the MIC values for both S29G and nisin A against *L. monocytogenes* LO28 and *S. mitis* UCC5001 were 6.28 and 8.38 mg/L, respectively. In contrast, nisin S29A exhibited enhanced specific activity against all Gram positive targets tested with MICs for *L. monocytogenes* LO28 and *S. mitis* UCC5001 being 3.14 and 4.19 mg/L, respectively.

It has previously been reported that the activity of nisin Z M21K is not enhanced against Gram positive targets [28]. Similarly, a *L. lactis* producer of M21K was found to display bioactivity comparable to that of a nisin A producing control when tested against the Gram positive targets *S. aureus* DPC 5245, MRSA ST528 and *S. agalactiae* ATCC13813 [29]. Here, MIC-based assays with M21K revealed that its specific activity against *L. lactis* HP, *S. aureus* RF122, *S. mitis* UCC5001 and *E. durans* is equal to that of nisin A but is reduced relative to nisin A against *B. cereus* DPC 6088 and DPC 6089.

### Specific Activities of Nisin A S29G, S29A, S29D and S29E against Gram Negative Organisms

Although nisin A has strong antibacterial activity against Gram positive organisms, the outer membrane (OM) of the Gram negative cell wall acts as a barrier for the cell, restricting the access of the peptide to the cytoplasmic membrane [44]. However, certain treatments which can disrupt the outer membrane of Gram negative bacteria can render them susceptible to nisin. Such treatments include chelating agents such as EDTA [45], sub-lethal heat, osmotic shock and freezing [46]. Furthermore, as noted above, a study involving mutagenesis of the hinge region of nisin Z uncovered two mutants N20K and M21K with enhanced antimicrobial activity against the Gram negative targets *Shigella, Pseudomonas* and *Salmonella* [28]. To assess the nisin S29 derivatives against a selection of Gram negative targets of particular foodborne significance, a modified agarose-based assay was utilized to assess the activity of purified nisin A and the nisin A S29G, S29A, S29D and S29E variants against *C. sakazakii* DPC 6440, *E. coli* 0157-H7 and *Salmonella enterica* serovar Typhimurium UK1. The nisin A M21K was also purified with a view to determining if it, like nisin Z M21K, showed enhanced anti-Gram negative activity. This analysis revealed that nisin A M21K exhibited improved activity against *C. sakazakii, E. coli* and *Salmonella* strains (Table 6). Corresponding studies revealed that the nisin S29E derivative exhibited increased activity against the *E. coli* and *Salmonella* strains only and that the nisin S29D was less active than nisin A against *E. coli* and *Salmonella* but exhibited activity comparable to that of nisin A against the *C. sakazakii* target (Table 6). In contrast, S29G and S29A displayed enhanced activity against all three Gram negative targets tested. Nisin A S29A consistently exhibited greatest potency in this regard (Table 6).

**Table 6:** Results of agarose gel diffusion assays against representative Gram negative strains.

STRAIN	NisinA mm	S29G mm(% wt/Pvalue)	S29A mm(% wt/P value)	S29D mm(% wt/P value)	S29E mm(% wt/P value)	M21K mm(% wt/P value)
*C. sakazakii* DPC 6440	9.77±0.17	**10.79**±0.16(110/0.002)	**11.65**±0.12(119/0.0002)	10.10±0.19 (103)	7.98±0.29 (81)	**10.51±**0.10(108/0.005)
*E. coli* 0157-H7	9.81±0.04	**11.10**±0.05(113/6E-06)	**11.50**±0.05(117/3E-06)	8.27±0.08 (84)	**10.54**±0.19(107/5E-05)	**10.80±**0.01(110/0.01)
*Salmonella enterica* serovarTyphimurium UK1	9.88±0.11	**10.94±**0.24(111/0.008)	**11.29±**0.23(114/0.003)	8.22±0.16 (83)	**10.77±**0.07(109/0.007)	**10.75±**0.12(109/0.008)

Results from agarose gel diffusion assays of nisin and S29 variants using purified peptide (60 µM) against the Gram negative strains *C. sakazakii* DPC 6440, *E. coli* 0157-H7 and *Salmonella enterica* serovar Typhimurium UK1. Values are the mean of triplicate agarose gel diffusion assays. All values in bold reached statistical significance compared to the nisin control (Student’s t-test: P<0.05).

To further confirm the specific activity of the peptides, and to ensure that these results were not a result of improved solubility in solid agar, broth-based MIC determination assays were also carried out using purified peptides against the same Gram negative targets. The results closely matched the patterns obtained using the agarose-based diffusion assays. More specifically, nisin A M21K exhibited a two-fold increase in specific activity compared to nisin A against all the targets tested (Table 7). Nisin A S29E was more active than nisin A against *E. coli* (25.14 mg/L and 50.28 mg/L respectively) and *Salmonella* (50.28 mg/L and >100 mg/L respectively) but not *C. sakazakii*, while nisin A S29D was more active than nisin A against *C. sakazakii* only (6.28 mg/L and 12.57 mg/L, respectively; Table 7). However, both S29G and S29A were two fold more potent against all three targets. These results establish that S29G and S29A variants differ from all nisin derivatives generated to date in that they exhibit enhanced activity against both Gram positive and Gram negative targets. It was also notable that S29A was the only derivative superior to nisin A against all strains utilized in this study.

**Table 7:** Minimum inhibitory concentrations of nisin derivatives against representative Gram negative strains.

STRAIN	NisinA mg/L (µM)	S29G mg/L (µM)	S29A mg/L (µM)	S29D mg/L (µM)	S29E mg/L (µM)	M21K mg/L (µM)
*C. sakazakii* DPC 6440	12.57(3.75)	**6.28(1.875)**	**6.28(1.875)**	**6.28(1.875)**	12.57(3.75)	**6.28(1.875)**
*E. coli* 0157-H7	50.28 (15)	**25.14(7.5)**	**25.14(7.5)**	>100(>30)	**25.14(7.5)**	**25.14(7.5)**
*Salmonella enterica* serovarTyphimurium UK1	>100(>30)	**50.28 (15)**	**50.28 (15)**	>100(>30)	**50.28 (15)**	**50.28 (15)**

Minimum inhibitory concentration assays of purified nisin wild type and the derivatives S29G, S29A, S29D, S29E and the hinge variant M21K against the Gram negative strains *C. sakazakii* DPC 6440, *E. coli* 0157-H7 and *Salmonella enterica* serovar Typhimurium UK1. Results are expressed as the mean of triplicate assays.

Finally, while MIC analyses can demonstrate the increased specific activity of a bioengineered peptide, their end point nature does not provide information regarding the relative bactericidal activity of peptides. To address this, the ability of S29G and S29A to kill Gram negative targets was tested and compared with that of nisin A.

**Figure 4:**
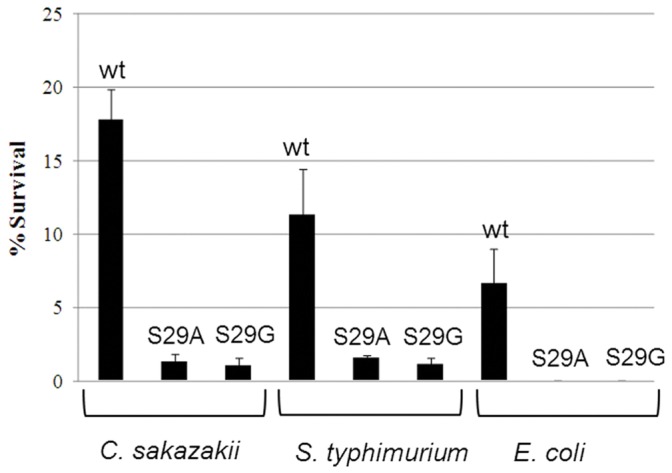
Kill curve analysis of strains *C. sakazakii* DPC 6440, *S. typhimurium* UK-1 and *E. coli* in 33mg/L respectively of nisin A, S29A and S29G.

Using purified peptide in each case, 10 mM sodium phosphate buffer washed *C. sakazakii, E. coli* and *Salmonella* strains were diluted to a final concentration of 2×10^5^ cfu ml^−1^ and were exposed to nisin A, S29G and S29A at a concentration of 33 mg/L. Following incubation at 37°C for 1 hour, bacterial growth was monitored through plate counts. In all instances the novel derivatives showed greater activity than wildtype nisin A (Fig. 4).

**Figure 5:**
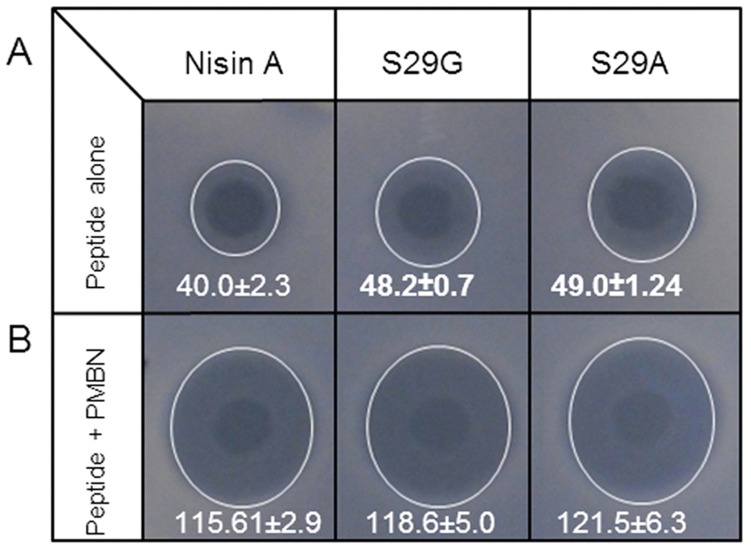
Activity of purified peptides against *C. sakazakii* DPC 6440. Activity of purified peptides of nisin A, S29G and S29A (30 µM) against *C. sakazakii* DPC 6440 as determined by agarose gel diffusion assay using (A) peptide alone and (B) peptide in combination with polymyxin B nonapeptide (PMBN) at a concentration of 20 µg/ml. Results are expressed as total area of inhibitory zone expressed in mm^2^. Values in bold reached statistical significance compared to the nisin control (Student’s t-test: P<0.05).

Assays with the polymyxin B nonapeptide (PMBN) were carried out in order to gain an insight into the basis for the enhanced activity of these derivatives against Gram negative targets. Polymyxin B (PMB) is a cyclic lipodecapaptide produced by bacteria of the genus *Paenibacillus* that acts primarily on the Gram negative cell wall [47], leading to rapid permeability changes in the cytoplasmic membrane and ultimately cell death. Polymyxin B nonapeptide (PMBN) lacks the fatty acid tail of PMB and, although it exhibits poor antimicrobial activity, it retains the ability to effect significant permeabilization of the OM, thus rendering Gram-negative bacteria susceptible to various hydrophobic antibiotics [48]. Notably, nisin A and polymyxin B have been shown to be more effective against Gram negative bacteria when used in combination than when either is used alone [49]. We sought to determine if the nisin derivatives S29G and S29A were also more potent than nisin A against Gram negative bacteria in which the OM no longer functions as an impenetrable barrier. To that end, *C. sakazakii* DPC 6440 cells were treated with purified nisin A, S29G and S29A peptides alone (30 µM) or in combination with PMBN at a concentration of 20 µg/ml and determined the antimicrobial activity by agarose gel diffusion assay (Fig. 5). As was previously observed, the S29G and S29A derivatives exhibited enhanced potency compared to nisin A when used alone (48.2±0.7 [P = 0.01], 49.0±1.24 [P = 0.009] and 40.0±2.3 respectively) (Fig. 5). When used in combination with PMBN, a substantial synergistic effect was clearly evident (Fig. 5) but in this instance no significant difference in efficacy was observed between nisin A or either of the derivatives S29A or S29G. Thus the enhanced activity of S29A and S29G over nisin A is only evident in situations where the OM is intact. These results suggest that the enhanced activity of the nisin derivative peptides against Gram negatives is as a result of an increased ability to traverse the OM relative to the nisin A peptide.

## Discussion

The ribosomally synthesised nature of lantibiotics and the consequent ability to conduct comprehensive bioengineering strategies provides tremendous potential for the development of more effective antimicrobials for food and medical applications. In this study, the screening of a randomly mutated bank of nisin derivatives produced a variant with superior activity against the strain *S. aureus* SA113. The increased efficacy resulted from a single mutation located at serine 29, within the C-terminal of nisin. The importance of Serine 29 for activity has previously been noted when Chan *et al* reported that the removal of five or nine residues from the C-terminal residues leads to a 16 fold or 110 fold decrease in bactericidal potency compared with that of intact nisin, respectively [35]. Additionally, Sun *et al* reported that nisin 1–28 also showed a 100 fold reduced inhibitory activity against *L. lactis* MG1363 [36]. These data pointed to an important role for Serine 29 in the activity of nisin and as a consequence, complete saturation mutagenesis was undertaken to determine the impact on nisin activity by substituting serine 29 with all the other available 19 standard amino acids. The strategy proved successful in that three more derivatives, S29A, S29D and S29E displayed enhanced activity against a range of bacterial targets. It is important to note that this improved activity was strain variable, providing further evidence that nisin derivatives can be generated with distinct target specificities. For example, studies with K22T (nisin T) revealed it to be more potent than nisin A against veterinary isolates of *S. aureus* and *S. agalactiae* [30] but not *Listeria monocytogenes,* while N20P (nisin P) is also striking by virtue of the target specific nature of its enhanced activity [29]. Similarly, S29D and S29E displayed improved activity against a distinct number of species, being particularly active against lactococci (*Lactococcus lactis* HP and *Lactococcus lactis* MG1363). In contrast, S29A was more potent than nisin A against all Gram positive and Gram negative bacterial targets.

While nisin was first approved for use in 1969, its use is likely to increase in the coming years due to the increased customer demand for minimally processed foods lacking artificial or chemical preservatives. A major concern in food safety is the transmission of pathogenic enterobacteriaceae (*Salmonella* spp, *E. coli* 0157:H7, *Shigella* spp) due to their major roles in foodborne illness [8]. While nisin is a potent anti-Gram positive inhibitor, its activity against Gram negative bacteria is poor. However, nisin can be used in combination with other synergistic preservation methods (known as hurdle technology), such as organic acids, low pH, high salt concentrations, chelating agents, modified atmosphere packaging, high hydrostatic pressure and thermal treatments, to enhance anti-Gram negative activity [8]. The superior activity of nisin A S29A compared to nisin A against Gram negatives, together with its enhanced activity against all Gram positive targets, suggests that S29A could find applications as a food preservative.

Nisin is also used in the veterinary industry and has potential as a clinical antimicrobial. Bovine mastitis is the cause of significant economic loss to dairy operations. Annual losses are presently estimated to be approximately $2 billion in the US alone [50]. Nisin A is already employed commercially as an anti-mastitis product in the form of Wipe Out®, and an intramammary infusion product Mast Out®, that are being developed as alternatives to traditional antibiotics. Indeed, the Center for Veterinary Medicine of the FDA has recently declared favourably on the application of nisin for the intramammary treatment for subclinical mastitis. More importantly, cattle would not be subject to a zero milk discard and a zero meat withhold as a consequence of treatment. Thus the existence of bioengineered nisin derivatives that consistently exhibit enhanced activity against mastitis associated pathogens such as the *S. agalactiae* and *S. aureus* RF122 strains utilized in this study is noteworthy. Furthermore, *Escherichia coli* can cause inflammation of the mammary gland in dairy cows around parturition and during early lactation with striking local and sometimes severe systemic clinical symptoms [51]. The bacterium invades the udder through the teat canal and may cause several cases of death per year in the most severe cases. The enhanced nature of S29A and S29G against Gram negative species such as *E. coli* as well as the major mastitis-associated Gram positive species, implies that these derivatives could also reduce the potential for economic loss as a result of their increased potency and broader target range. Indeed, the synergism of nisin A in combination with the polymyxin B nonapeptide reported here would suggest a potential use for this potent combination to control bovine mastitis and, potentially, other veterinary and clinical infections. However, further study is required to establish the mechanistic basis for the enhanced activity of the S29G and S29A derivatives relative to nisin A. In particular, studies will focus on further investigating the importance of the OM with respect to their superior activity against Gram negative bacteria, for which nisin is usually considered ineffective.

In conclusion, it is apparent that altering residue 29 of nisin A can result in the generation of variants with enhanced antimicrobial activity. In some instances this enhancement varies depending on the target microorganism but in other cases, particularly nisin A S29A and, to a lesser extent, S29G, this enhancement is consistent across a wide range of targets. The fact that this enhancement is apparent against Gram positive and Gram negative targets is particularly novel. Further efforts will focus on determining the mechanistic basis for these enhancements and an assessment of how well these peptides perform in food and *in vivo*.
